# Beyond demographic tables: integrating data quality in clinical trial representativeness

**DOI:** 10.3389/fdgth.2026.1800079

**Published:** 2026-05-07

**Authors:** João Gregório, Agnieszka Lemanska, Bartlomiej Cieszynski, Nina Perić, Moulham Alsuleman, Michael Chrubasik, Paul Duncan

**Affiliations:** 1 Informatics, Data Science Department, National Physical Laboratory, Glasgow, United Kingdom; 2 Imperial Clinical Trials Unit, School of Public Health, Imperial College London, London, United Kingdom; 3 Informatics, Data Science and AI Department, National Physical Laboratory, Teddington, United Kingdom

**Keywords:** clinical trial representativeness, completeness dimension, coverage metric, data quality framework, demographic similarity, electronic health records, fitness-for-purpose assessment, Jensen–Shannon distance

## Abstract

**Introduction:**

Clinical trial representativeness is essential for ensuring that study findings generalise to target treatment populations. Current assessment approaches rely on subjective demographic comparisons that lack standardisation and fail to account for data quality. Existing data quality frameworks assess completeness at the dataset level as the availability of required data values but do not address cohort-specific suitability for representativeness analysis. This study proposes that for clinical trial datasets, the completeness dimension should be interpreted through two complementary aspects: demographic coverage (the degree to which trial demographics represent the target population) and dataset completeness (the availability of required demographic data for analysis). Missing demographic data can compromise similarity assessments, yet no standardised approach exists to integrate both aspects of completeness into representativeness evaluation.

**Methods:**

This work introduces a quantitative framework that operationalises this dual interpretation of completeness. Coverage is measured using Jensen–Shannon Distance across demographic distributions, while dataset completeness quantifies the percentage of valid data available. These metrics are aggregated through clinically informed weights into a single suitability score. The framework was validated using simulated trial cohorts sampled from the MIMIC-III database and compared against a target sepsis population of 1,315 patients extracted from the same dataset. The validation employed simple random sampling with varying cohort sizes to simulate Phase II/III single-centre trial scenarios.

**Results:**

Results showed that demographic alignment drives overall suitability. Correlation analysis confirmed that coverage and completeness vary independently, validating the composite metric approach. Suitability scores ranged from 86.5% to 91.6% (mean 89.1%), demonstrating the framework’s ability to discriminate between cohorts. Cohort size showed no correlation with suitability (r=0.08, p=0.42), indicating that high representativeness can be achieved in smaller trials. Patient weight and language showed the lowest completeness, reflecting dataset limitations that can be mitigated through feature weighting.

**Discussion:**

Interpretability and reproducibility suggest potential for integration into trial design workflows, supporting evidence-based enrolment strategies and transparent regulatory evaluation. By providing systematic, quantifiable metrics, this work advances clinical trial quality assessment beyond qualitative comparisons and establishes a foundation for systematic evaluation of trial representativeness.

## 1 Introduction

Clinical trials are among the primary methods for evaluating new medical devices prior to regulatory approval. Generalisation of trial findings to real-world populations is critical for clinical adoption and evidence-based decision-making. Trial protocols typically impose inclusion and exclusion criteria that shape cohort composition, often resulting in demographic differences from the intended treatment population in terms of age, gender, ethnicity, and other characteristics [1–4].

Current practice for assessing trial representativeness relies on manual comparison of demographic tables, where descriptive statistics are inspected to identify discrepancies between trial cohorts and reference populations [4–6]. This approach is subjective, time-consuming, and lacks standardised metrics for quantifying overall representativeness. Automated methods such as propensity score matching exist [7], but they generally focus on single variables, require complex modelling assumptions, and rarely integrate multiple demographic dimensions or account for data quality. Machine learning approaches have also been explored for predicting trial participation [3, 8], yet these methods often lack interpretability for trial design decisions. Information-theory measures such as Jensen-Shannon Distance (JSD) have been applied in healthcare for comparing distributions [9–13], but their use for multi-dimensional demographic similarity in clinical trial assessment remains limited. Furthermore, existing data quality frameworks emphasise completeness and other dimensions [14–17], but they generally address dataset-level quality rather than cohort-specific suitability. These gaps motivate the need for a transparent, quantitative framework that integrates representativeness and data quality considerations.

To address these limitations, this work applies established data quality principles, with emphasis on the completeness dimension, which is critical for reliable representativeness assessment. Completeness is defined as “the degree to which subject data associated with an entity have values for all expected attributes and related entity instances in a specific context of use” [16]. While this definition emphasises data availability at the dataset level, it does not address whether collected data adequately represents the intended study population—a critical consideration for clinical trials where cohort composition directly affects generalizability.

This work proposes that for clinical trial datasets, completeness can be interpreted through two complementary aspects: *coverage*, which measures how well the trial cohort reflects the demographic characteristics of the target population, and *dataset completeness*, which quantifies the availability of required demographic data for analysis. This dual interpretation recognizes that a trial with complete demographic data for all enrolled patients may still fail to represent the target population if key demographic groups are systematically excluded. Conversely, a demographically representative cohort with substantial missing data cannot support reliable similarity assessment.

Obtaining suitable reference data is non-trivial. Electronic health record databases offer potential sources but require extensive preprocessing to resolve fragmentation, non-standard terminologies, and missing data patterns [14]. In this work, a standardised dataset derived from Medical Information Mart for Intensive Care III (MIMIC-III) [18, 19] is used to enable systematic analysis and to support the development of a transparent, reproducible framework for assessing trial cohort representativeness.

The proposed framework formalises two metrics: a *coverage metric*, computed using JSD across seven demographic and sociodemographic characteristics (age, gender, ethnicity, weight, language, religion, and marital status), and a *completeness metric*, which quantifies the proportion of valid data available for analysis. While variables such as ethnicity, language, religion, and marital status can serve as proxies for socioeconomic factors that influence health outcomes, they do not constitute formal deprivation indices such as the Index of Multiple Deprivation or area-level socioeconomic indicators. These metrics are aggregated through clinically informed weights into a single suitability score that provides an interpretable measure of representativeness.

The framework is validated using simulated trial cohorts compared against a target sepsis population extracted from MIMIC-III. This work builds upon a previously established data standardisation pipeline [20] and extends it by proposing an interpretation of the completeness data quality dimension in the context of clinical trial datasets that includes both population coverage and dataset completeness. It also provides a proof-of-concept methodology demonstrating the feasibility of systematic representativeness assessment.

## 2 Methodology

**Figure 1:**
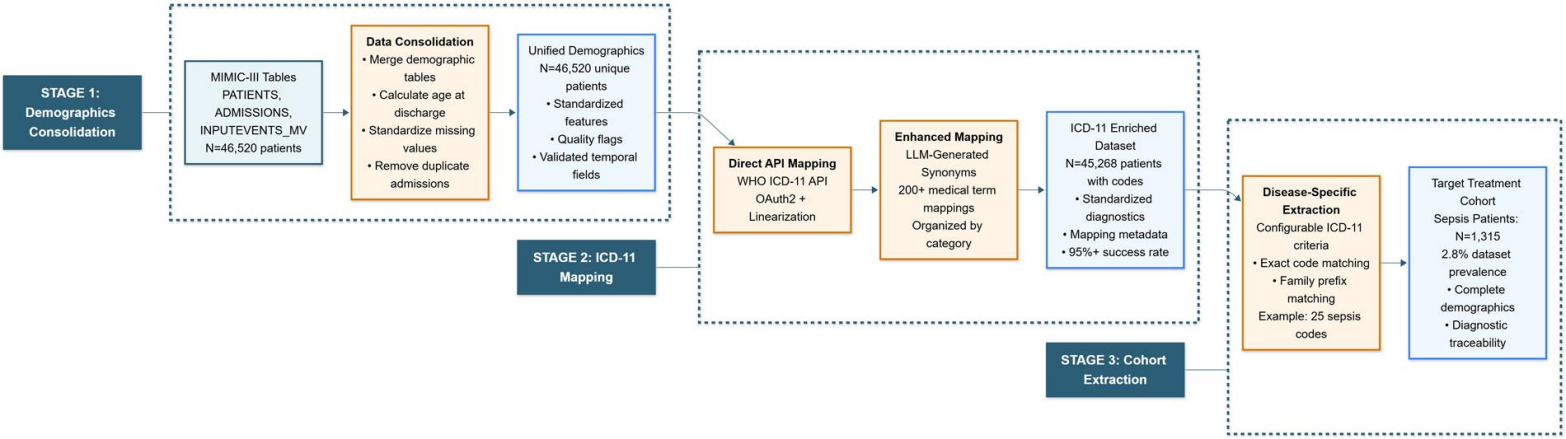
The diagram shows three sequential stages in the data preparation pipeline: Stage 1 (Demographics Consolidation) merges patient tables from MIMIC-III, calculates age at discharge, and standardises missing values to produce a unified demographic dataset. Stage 2 (ICD-11 Mapping) enriches diagnostic information through direct WHO API mapping and enhanced synonym-based mapping using language models, resulting in a standardised ICD-11 coded dataset. Stage 3 (Cohort Extraction) applies configurable ICD-11 criteria to identify disease-specific cohorts (e.g., sepsis), ensuring complete demographic and diagnostic traceability. Dashed boxes delimit each stage, highlighting the transformation from raw clinical data to structured cohorts suitable for downstream analysis.

The methodology consists of two primary components: a data extraction, transformation, and loading (ETL) pipeline (Stages 1–3) [20] that prepares clinical data for analysis, and a novel representativeness assessment framework (Stage 4) that quantifies how well trial cohorts represent target treatment populations. The workflows for stages 1–3 and stage 4 are shown in Figure 1. All computational workflows were implemented in Python using the SciPy ecosystem as the primary platform for scientific computing [21].

The dataset used in this work is the Medical Information Mart for Intensive Care III (MIMIC-III) database version 1.4, a publicly accessible repository containing anonymised health records from 46,520 adult patients admitted to critical care units at Beth Israel Deaconess Medical Center, USA between 2001 and 2012 [18]. It contains clinical information, including patient demographics (age, gender, ethnicity, language, religion, and marital status), physiological measurements, laboratory results, diagnostic codes, medications, and mortality outcomes. All data is anonymised through structured data cleansing, date shifting, and removal of protected health information from free-text fields. Dates were shifted forward by patient-specific random offsets to preserve temporal intervals while maintaining anonymity.

### 2.1 Data preparation pipeline: stages 1–3

The ETL pipeline transforms raw MIMIC-III clinical data into standardised, research-ready cohorts through three sequential stages.

Stage 1 aggregates fragmented demographic data from multiple MIMIC-III tables into a unified dataset. Patient age is calculated using discharge time as the primary method with fallback to admission time or time of death for deceased patients. Multiple admissions for the same patient are resolved by retaining only the first admission, ensuring each individual appears once. Missing value representations are standardised under common designations. The output is a unified demographics table with validated temporal fields and quality flags.

Stage 2 standardises non-uniform MIMIC-III diagnostic terms to International Classification of Diseases 11th Revision (ICD-11) codes [22, 23] via the WHO’s official API. Initial direct mapping attempts exact term matching. Unmapped terms undergo enhanced mapping using Large Language Model (LLM) generated medical synonyms [24] (e.g., “CHF” → “congestive heart failure”, “heart failure”, “cardiac failure”). The synonym dictionary contains over 200 medically validated term mappings organised by clinical category. The output is the demographics table enriched with standardised ICD-11 codes, mapping metadata, and traceability information.

Stage 3 identifies target treatment populations using configurable ICD-11 code criteria. Both exact code matching and family-based prefix matching capture diagnostic variants. In this work, sepsis patients were extracted using 25 ICD-11 codes covering systemic sepsis, neonatal sepsis, organism-specific variants, and ten code families. The output is a dataset containing a sepsis treatment cohort with complete demographic profiles and diagnostic traceability, serving as the target population for assessing coverage and completeness of hypothetical clinical trial cohorts in stage 4.

### 2.2 Trial cohort representativeness assessment: stage 4

Stage 4 formalises a data quality assessment framework for quantifying how well clinical trial cohorts represent target treatment populations. To validate the methodology, 100 simulated trial cohorts were generated from the non-sepsis population using simple random sampling. Simple random sampling was chosen to isolate the effects of demographic similarity and data completeness, providing a controlled validation environment. Cohort sizes were drawn from a Gaussian distribution (μ=60, σ=13, clipped range: 20–100 patients), reflecting typical Phase II/III single-centre enrolment scenarios. Each cohort was sampled without replacement using different random seeds to ensure variation across simulations. Seven demographic dimensions were assessed across all cohorts:

- **Age Groups:** Six categories reflecting distinct clinical considerations: Neonate/Infant (0–1 years), Child/Adolescent (1–18 years), Young Adult (18–40 years), Middle-aged (40–65 years), Older Adult (65–80 years), and Elderly (80+ years).
- **Weight Groups:** Five categories based on clinical dosing significance: Underweight (<50 kg), Normal (50–70 kg), Overweight (70–90 kg), Obese (90–120 kg), and Severely Obese (>120 kg).
- **Categorical Variables:** Gender (Male/Female), Ethnicity (41 categories consolidated to seven major groups: White, Black/African American, Hispanic/Latino, Asian, Other, Multiple, Unknown), Religion (20 categories), Language (75 categories), and Marital Status (seven categories).

Age categories were defined based on distinct clinical and pharmacological considerations: neonatal/infant physiology differs substantially from older children; the 1–18 year range reflects pediatric dosing protocols; young adults (18–40) represent the healthy baseline population typically excluded from age-related comorbidity considerations; middle-aged adults (40–65) mark the onset of chronic disease prevalence; older adults (65–80) represent the primary demographic for most clinical interventions; and elderly patients (80+) require specialised geriatric considerations.

While language, religion, and marital status received lower weights (0.05 each) reflecting their variable relevance across therapeutic contexts, they were retained to demonstrate the framework’s flexibility in accommodating diverse representativeness priorities depending on study objectives.

#### 2.2.1 Coverage metric

Dataset coverage is quantified by demographic similarity between trial and target populations using JSD. For each demographic feature f, probability distributions are computed from trial cohort and target population category counts:

$$p_{\text{trial}}^{f}(c)=\frac{n_{\text{trial}}^{f}(c)}{\sum_{c^{\prime }}n_{\text{trial}}^{f}(c^{\prime })},\;p_{\text{target}}^{f}(c)=\frac{n_{\text{target}}^{f}(c)}{\sum_{c^{\prime }}n_{\text{target}}^{f}(c^{\prime })}$$ (1)

where c indexes categories within feature f. “Trial” and “target” represent the clinical trial and target treatment populations respectively. The midpoint distribution is calculated as:

$$M^{f}(c)=\frac{\;p_{\text{trial}}^{f}(c)+p_{\text{target}}^{f}(c)}{2}$$ (2)

Kullback–Leibler divergences [25] from each distribution to the midpoint are computed:

$$KL(p_{\text{trial}}^{f}\|M^{f})=\sum_{c}p_{\text{trial}}^{f}(c)\cdot \log\left(\frac{\;p_{\text{trial}}^{f}(c)}{M^{f}(c)}\right)$$ (3)

with analogous calculation for $KL(p_{\text{target}}^{f}\|M^{f})$. To ensure numerical stability when probability values approach zero, a small constant ($\epsilon =10^{-10}$) is added to all probability terms before computing logarithms, preventing undefined operations in the KL divergence calculation. The JSD is Menéndez et al. [11]:

$${\text{JSD}}^{f}=\sqrt{\frac{1}{2}\cdot KL(p_{\text{trial}}^{f}\|M^{f})+\frac{1}{2}\cdot KL(p_{\text{target}}^{f}\|M^{f})}$$ (4)

Converting to a coverage percentage where higher values indicate better representativeness:

$${\text{Coverage}}^{f}=(1-{\text{JSD}}^{f})\times 100\%$$ (5)

Feature-level coverage scores are aggregated using weights reflecting regulatory priorities and pharmacological significance:

$${\text{Coverage}}_{\text{overall}}=\sum_{\;f=1}^{7}w_{f}\cdot {\text{Coverage}}^{f}$$ (6)

where weights are: AGE_GROUP (0.30), GENDER (0.25), ETHNICITY (0.20), WEIGHT_GROUP (0.10), RELIGION (0.05), LANGUAGE (0.05), MARITAL_STATUS (0.05). These weights reflect clinical and regulatory convention, prioritising age, gender, and ethnicity as primary determinants of pharmacological response and trial generalisability. They represent informed design choices rather than empirically derived values, and their systematic validation remains a direction for future work.

#### 2.2.2 Completeness metric

Dataset completeness is quantified via the proportion of valid demographic data, addressing how missing information impacts trial validity. For each feature f:

$${\text{Completeness}}^{f}=\frac{n_{\text{complete}}^{f}}{n_{\text{total}}}\times 100\%$$ (7)

where $n_{\text{complete}}^{f}=n_{\text{total}}-n_{\text{missing}}^{f}-n_{\text{unknown}}^{f}$. Both null values and explicit unknown designations (“UNKNOWN”, “PATIENT DECLINED”, “UNABLE TO OBTAIN”) are counted as incomplete data.

Feature-level completeness scores are aggregated with weights reflecting analytical importance:

$${\text{Completeness}}_{\text{overall}}=\sum_{\;f=1}^{7}w_{f}\cdot {\text{Completeness}}^{f}$$ (8)

where the same feature weights as coverage are applied (see Equation 6).

#### 2.2.3 Combined metric

The suitability of a trial cohort for representing a target treatment population is represented by a combined metric:

$$\text{Suitability}=0.60\cdot {\text{Coverage}}_{\text{overall}}+0.40\cdot {\text{Completeness}}_{\text{overall}}$$ (9)

The weights assigned to the suitability metric in this work are mainly illustrative, reflecting the nuances of real-world scenarios where coverage and completeness impact differently. These design choices introduce a degree of subjectivity that is acknowledged as a limitation of the current framework. Future work should consider empirical or expert-informed approaches to weight derivation, such as the Delphi method [26] or the Best-Worst Method [27], to provide stronger justification for the chosen values. Coverage was given a higher weight (60%) than completeness (40%) due to representative enrolment being more relevant for ensuring generalisability. Missing data values can often be mitigated through statistical methods, whereas demographic misalignment fundamentally limits external validity.

To validate the composite metric approach, the independence of coverage and completeness was assessed using both Pearson’s correlation coefficient (measuring linear relationships) and Spearman’s rank correlation coefficient (measuring monotonic relationships) across all simulated cohorts. Independent metrics justify combining them into a single score, as high coverage does not guarantee high completeness and vice versa. If these metrics were highly correlated (|r|>0.7), one metric would be sufficient; weak correlation (|r|<0.3) confirms they capture distinct aspects and validates the combined metric approach.

#### 2.2.4 Worked example

Assuming a hypothetical trial cohort has the following coverage and completeness scores relative to the target treatment population:

- **Coverage scores:** Gender (98%), Age Group (91%), Ethnicity (89%), Weight Group (86%), Religion (82.50%), Language (79%), Marital Status (85%) → Overall: 90%
- **Completeness scores:** Age (89%), Ethnicity (95%), Gender (100.00%), Weight (62%), Religion (80%), Language (56%), Marital Status (88%) → Overall: 88.1%

both obtained by the application of Equations 6 (coverage) and 8 (completeness) respectively. Applying these values to Equation 9 yields a suitability score of 89.2%.

In this scenario, the trial cohort shows strong demographic representativeness alongside good data completeness. The documented limitations of the dataset (such as the 38% missing weight data) provide additional context that can support clinical decision-making. By making these limitations explicit, practitioners gain a more complete understanding of the trial population, which enables more informed judgements about the applicability of the study to individual treatment cases.

## 3 Results

**Figure 2:**
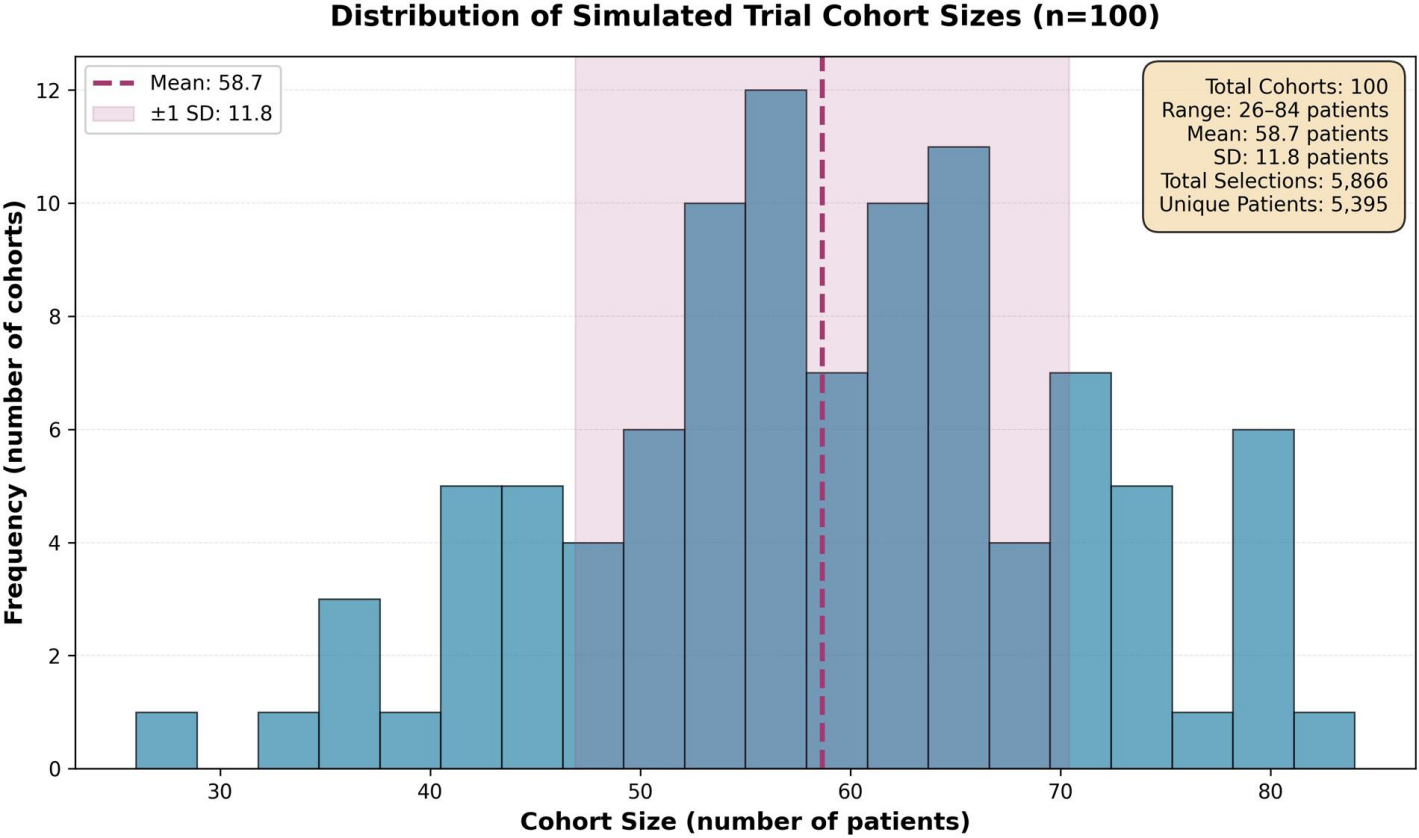
Distribution of simulated trial cohort sizes. Histogram showing cohort sizes across 100 simulated trials (mean = 58.7, SD = 13.2, range: 26–84 patients). The sampling process selected 5,866 total patients with 5,395 unique individuals (91.9% unique selection rate), indicating quasi-independent cohorts suitable for comparative analysis. Dashed line indicates mean cohort size; shaded region represents ±1 standard deviation.

The sizes of the simulated cohorts followed a Gaussian distribution (mean = 58.7, SD = 13.2, range: 26–84 patients), as shown in Figure 2. The observed distribution closely matched the specified Gaussian parameters with slight deviation due to finite sampling. The sampling process resulted in 5,866 total patient selections with 5,395 unique patients used, yielding a 91.9% unique selection rate with minimal overlap between cohorts. Each cohort was assessed against the target sepsis treatment population of 1,315 patients across seven demographic dimensions.

### 3.1 Overall and feature-level performance

Overall performance metrics are summarised in Table 1. Suitability scores ranged from 86.5% to 91.6%, with a mean of 89.1%. Coverage and completeness exhibited similar ranges, from 82.4% to 91.9% and 88.3% to 94.7%, respectively. Correlation between coverage and completeness was weak (r=−0.29, p<0.01), indicating that these dimensions vary independently.

**Table 1:** Performance across 100 trial cohorts.

Metric	Min (%)	Max (%)	Mean (%)	SD
Suitability	86.5	91.6	89.1	1.1
Coverage	82.4	91.9	87.5	1.9
Completeness	88.3	94.7	91.4	1.2

Minimum, maximum, mean, and standard deviation (SD) for Suitability (combined score), Coverage (demographic similarity), and Completeness (data quality). Suitability is calculated as a weighted aggregation of coverage and completeness (weights: 0.60 and 0.40 respectively).

Feature-level performance is shown in Table 2. Gender achieved the highest coverage (90.8%), while ethnicity and language were lowest (83.4% and 81.7%). Completeness was near-perfect for most features except patient weight (47.3%) and language (67.8%). High-weight features such as age (0.30), gender (0.25), and ethnicity (0.20) contributed most to overall suitability scores, whereas completeness issues in lower-weight features had limited impact.

**Table 2:** Feature-level coverage and completeness scores.

Feature	Weight	Coverage (%)	Completeness (%)
Gender	0.25	90.8	100.0
Age Group	0.30	86.7	95.2
Ethnicity	0.20	83.4	100.0
Weight Group	0.10	89.1	47.3
Religion	0.05	85.3	100.0
Language	0.05	81.7	67.8
Marital Status	0.05	88.6	100.0

Mean coverage and completeness percentages for each demographic feature across 100 trial cohorts, along with feature weight used in suitability calculation.

### 3.2 Extreme cases and cohort size

The two extreme cases observed among the 100 cohorts are compared in Table 3. The best-performing cohort (n=51) achieved 91.6% suitability, while the lowest (n=45) achieved 86.5%. Cohort size showed no meaningful relationship with suitability (r=0.08, p=0.42), coverage (r=0.06, p=0.55), or completeness (r=−0.18, p=0.07), suggesting that representativeness outcomes were not strongly influenced by sample size within the tested range.

**Table 3:** Case study: highest and lowest performing cohorts.

Cohort	Size (n)	Suitability (%)	Coverage (%)	Completeness (%)
Highest	51	91.6	91.0	92.5
Lowest	53	86.5	82.4	92.7

Comparison of two extreme cases observed among the 100 simulated cohorts. Suitability is the combined metric, while coverage and completeness represent the underlying components. Cohort size (n) is included for context.

**Figure 3:**
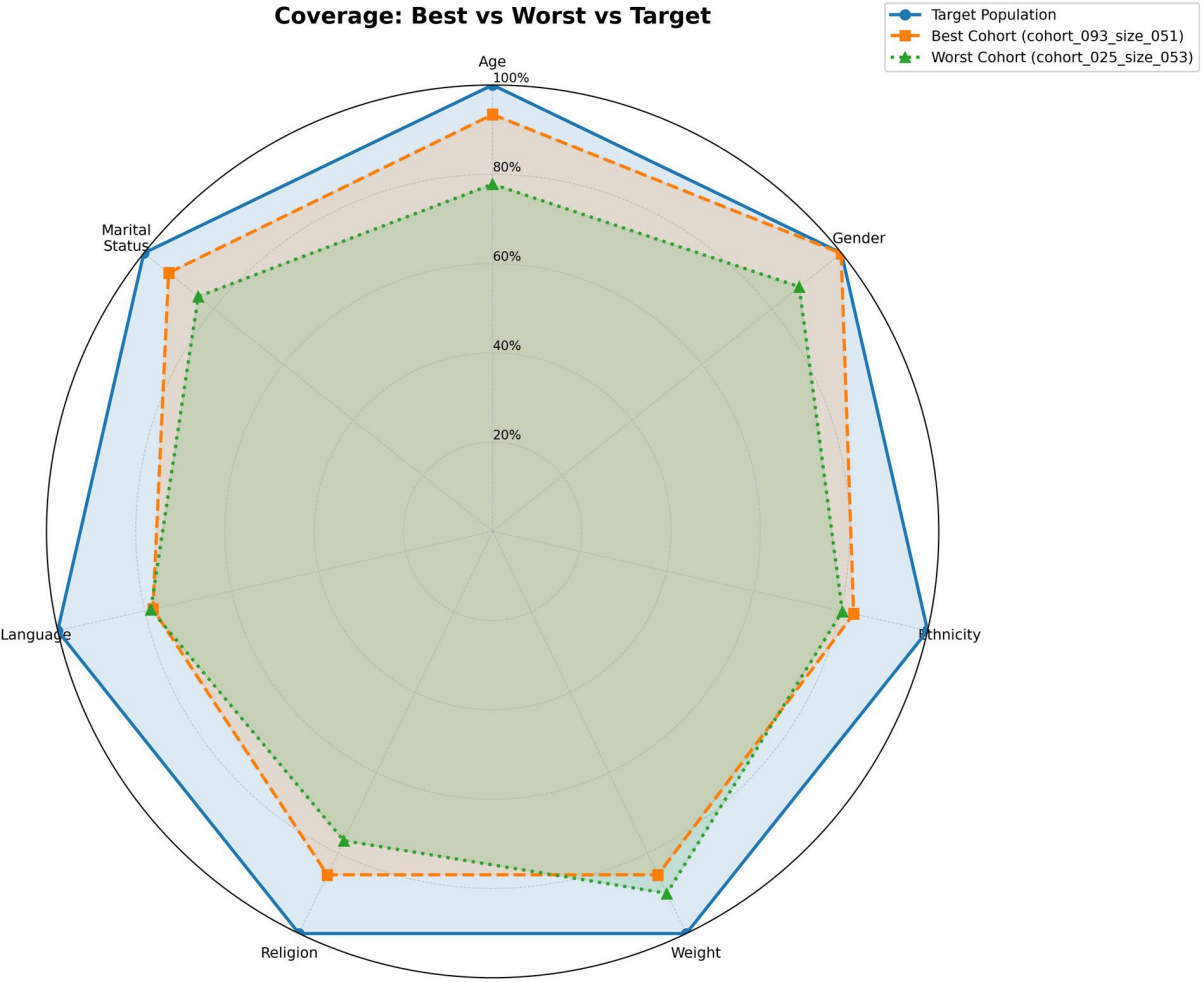
Coverage comparison across demographic features. Radar plot showing demographic similarity between trial cohorts and the target sepsis population across seven features. The target population (blue solid line) forms the outer boundary at 100% by definition. The best cohort (orange dashed, 91.6% overall suitability) maintains closer alignment particularly in high-weight features (age, gender, ethnicity), while the worst cohort (green dotted, 86.5% overall suitability) shows systematic gaps across all dimensions.

**Figure 4:**
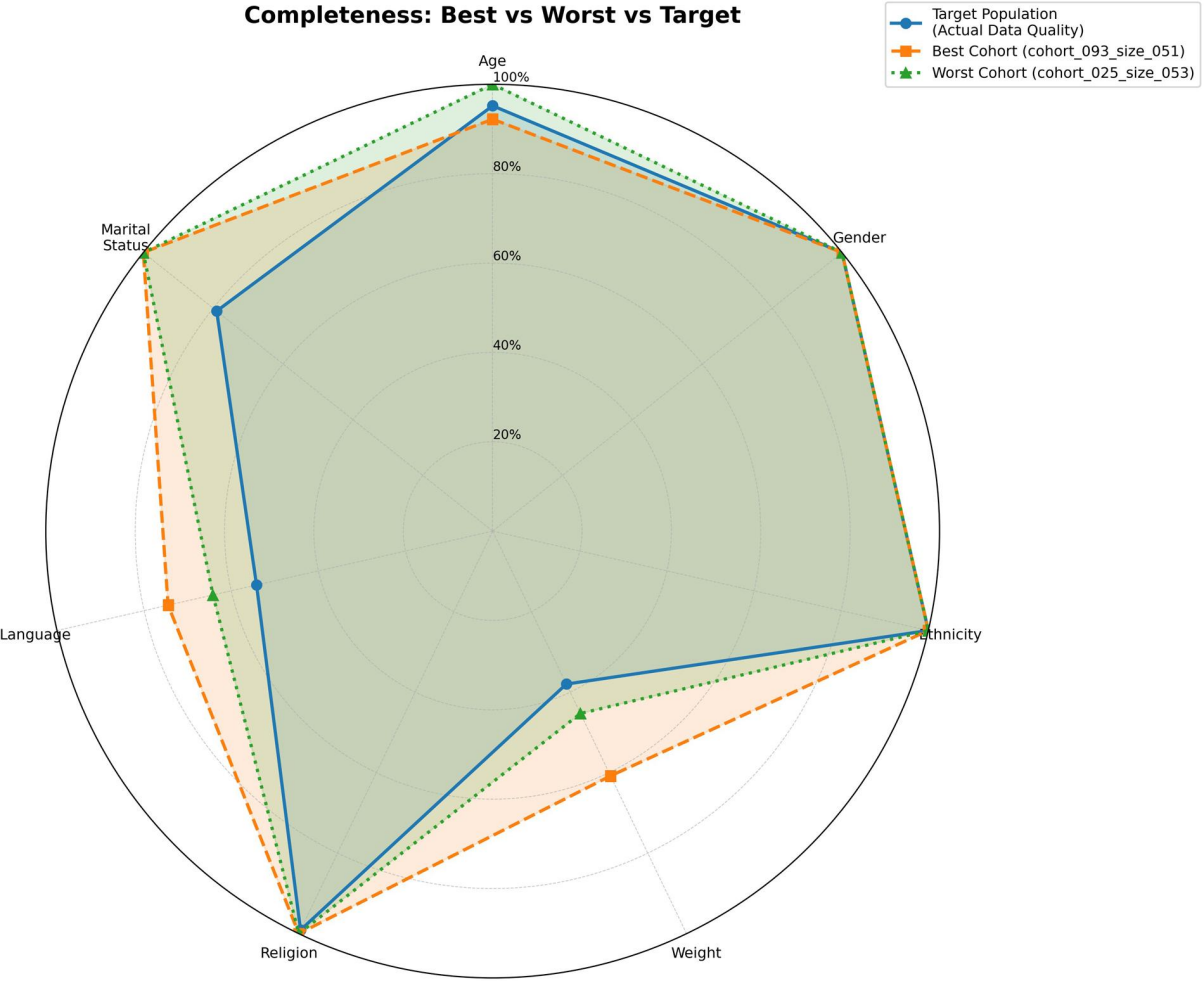
Completeness comparison across demographic features. Radar plot showing data quality (proportion of valid, non-missing values) for the target population and trial cohorts. The target population exhibits an irregular shape reflecting actual MIMIC-III limitations, with weight and language incompleteness while other features approach 100% completeness. Both trial cohorts track this profile closely, indicating that data quality issues are inherited from the source population rather than introduced through sampling.

**Figure 5:**
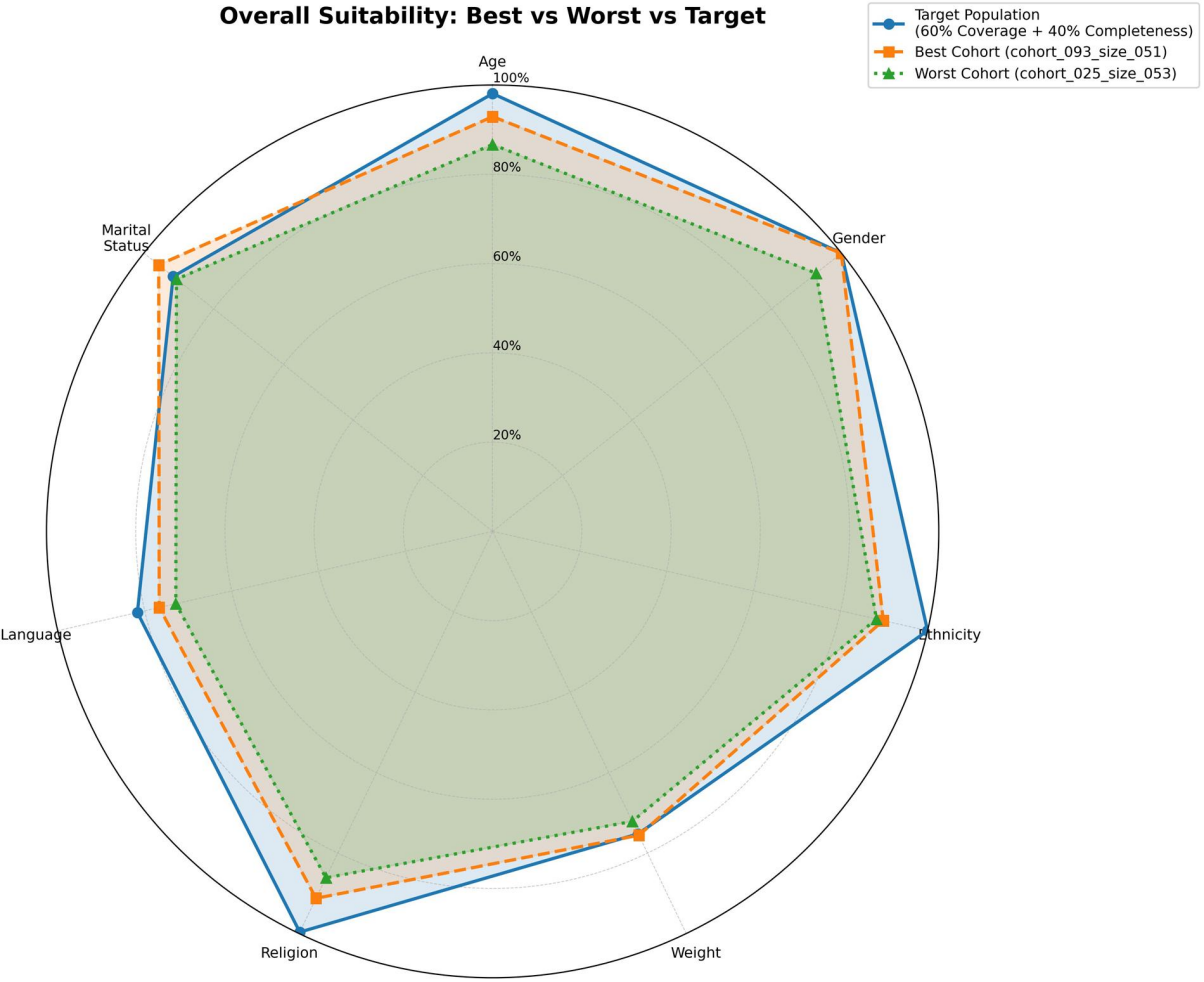
Overall suitability combining coverage and completeness. Radar plot showing composite suitability scores integrating demographic similarity (60% weight) and data quality (40% weight) across all features. The best cohort (orange dashed, 91.6%) maintains closer alignment across most features, while the worst cohort (green dotted, 86.5%) shows cumulative deficits particularly in age, and language.

Figures 3–5 provide radar plots comparing the target population with the highest- and lowest-performing cohorts across all seven demographic features. Figure 3 shows coverage profiles, where the best cohort aligns more closely to the target in high-weight features (age, gender, ethnicity) than the worst cohort. Figure 4 illustrates completeness patterns, highlighting incompleteness in weight and language across all profiles. Figure 5 integrates both dimensions into the composite suitability metric, showing how differences in coverage drive overall score variation.

### 3.3 Robustness checks

To assess the sensitivity of suitability scores to the design choices inherent in the weighting scheme, a new set of simulated cohorts was generated as part of the review process using identical sampling parameters and random seed to the original analysis (Gaussian distribution, μ=60, σ=13; mean size 58.7, range 26–84 patients, total selections 5,866). Because cohort sizes are determined solely by the random seed and sampling parameters, the size distribution is identical to the original set of cohorts. However, the patients assigned to each cohort depend on the row ordering of the source dataset at the time of generation, which differed between pipeline runs due to differences in the row ordering of the non-sepsis dataset produced by Stage 3. No changes were made to any pipeline script or parameters between runs; the difference is purely a consequence of the non-sepsis dataset being written to disk at a different time, resulting in a different internal row ordering. Since random sampling draws patients by position within the dataframe, identical seeds applied to differently ordered inputs produce cohorts of identical size but different patient composition. Consequently, cohorts with the same index label (e.g., C-092) contain different patients than in the original analysis and cannot be compared directly to those reported in Sections 3.1 and 3.2 or the associated figures (Figures 3–5).

Robustness checks were then conducted by re-scoring this regenerated cohort set under alternative weighting configurations. Two sets of perturbations were applied independently: variation of the feature weights used to aggregate coverage and completeness scores (Equations 6 and 8), with results shown in Table 4, and variation of the aggregation weights used to combine coverage and completeness into the suitability score (Equation 9), with results shown in Table 5. For each scenario, summary statistics and the identity of the highest- and lowest-performing cohorts within this regenerated set are reported.

**Table 4:** Suitability scores under alternative feature weight configurations.

Scenario	Mean (%)	SD	Min (%)	Max (%)	Best	Worst
Current (clinical)	85.1	1.33	81.8	88.3	C-092	C-091
Equal weights	82.1	1.61	78.3	86.0	C-092	C-064
Age-dominant	84.9	1.45	80.8	88.7	C-096	C-091

Aggregation weights fixed at 60% coverage and 40% completeness across all scenarios. The current (clinical) scenario applies weights of 0.30 (Age), 0.25 (Gender), 0.20 (Ethnicity), 0.10 (Weight), and 0.05 each for Religion, Language, and Marital Status. Equal weights assigns 1/7≈0.14 to each feature. Age-dominant assigns 0.50 to Age, with remaining weight distributed across other features.

**Table 5:** Suitability scores under alternative aggregation weight configurations.

Scenario	Mean (%)	SD	Min (%)	Max (%)	Best	Worst
Coverage-dominant (70/30)	84.5	1.46	80.6	87.9	C-092	C-091
Current (60/40)	85.1	1.33	81.8	88.3	C-092	C-091
Equal (50/50)	85.8	1.22	82.9	88.7	C-092	C-091

Feature weights fixed at current clinical values across all scenarios. Coverage-dominant assigns 70% to coverage and 30% to completeness; Equal assigns 50% to each. Cohort rankings (best and worst) remain stable across all three scenarios.

Under feature weight perturbation (Table 4), mean suitability scores varied between 82.1% (equal weights) and 85.1% (current clinical weights), reflecting the redistribution of weight toward lower-coverage features such as language and religion under the equal weights scenario. Cohort rankings were largely stable: the best-performing cohort was consistent across the current and equal weights scenarios (C-092), with C-096 emerging as best under the age-dominant scenario due to its superior age group alignment (83.2% vs. 79.1% for C-092). The worst-performing cohort was consistent across current and age-dominant scenarios (C-091), with C-064 identified as worst under equal weights.

Aggregation weight perturbation (Table 5) produced stable rankings across all three scenarios, with both the best- (C-092) and worst-performing (C-091) cohorts unchanged. The standard deviation narrowed progressively as completeness weight increased (1.46 at 70/30 to 1.22 at 50/50), consistent with completeness exhibiting less cross-cohort variation than coverage. Taken together, these results indicate that cohort rankings are robust to reasonable perturbations of the aggregation weights, and largely stable under feature weight variation, with rank changes under extreme configurations attributable to interpretable differences in feature-level alignment.

## 4 Discussion

This study demonstrates the feasibility of a systematic, quantitative framework for assessing clinical trial cohort representativeness using a dual interpretation of data quality completeness. By separating coverage (demographic similarity) from dataset completeness (data availability), the framework provides a structured approach to evaluating trial cohorts beyond traditional descriptive comparisons.

The results show that demographic alignment is the dominant driver of overall suitability under the adopted weighting scheme (60% coverage, 40% completeness). The 5.1 percentage-point gap between the highest and lowest performing cohorts was primarily attributable to coverage differences rather than completeness. This finding aligns with the conceptual emphasis on coverage as a prerequisite for generalisability: even when data completeness is high, demographic misalignment can substantially reduce suitability. Feature-level analysis reinforces this observation, with high-weight features such as age, gender, and ethnicity exerting the greatest influence on suitability scores. Completeness limitations in lower-weight features (weight and language) had limited impact, suggesting that weighting schemes can effectively mitigate the influence of systematic data quality constraints when these occur in features of lower clinical priority.

Visualisations highlight the structural limitations of the source dataset. Completeness profiles for both trial cohorts closely mirrored the target population, confirming that incompleteness patterns were inherited rather than introduced through sampling. Patient weight and language exhibited the largest gaps, reflecting known limitations in MIMIC-III [19]. While patient weight was used due to MIMIC-III data availability, future implementations should employ body mass index (BMI), which better captures metabolic risk and dosing considerations across diverse populations. These constraints underscore the importance of considering data quality when interpreting representativeness metrics: while demographic similarity can be quantified, the reliability of these assessments depends on the integrity of underlying data. The irregular suitability profile of the target population itself illustrates this point. This shows that representativeness assessment is not solely a function of cohort composition but also of data completeness, reinforcing the fitness-for-purpose paradigm in data quality frameworks [14, 16]. Despite perfect demographic self-similarity, missing data in patient weight and language reduced composite scores for these features. Although patient weight here had a reduced contribution to the overall score, for medical conditions where weight is more important (e.g., diabetes), a higher weighting may be applied and the suitability score would decrease accordingly. The incompleteness of both these features were mitigated by their reduced contributions to the overall completeness score, and, by consequence, to the overall suitability score. This demonstrates the mitigating effect of feature prioritisation on systematic data quality constraints.

Compared with existing approaches such as propensity score matching [7] and machine learning-based eligibility prediction [3, 8], the proposed framework offers interpretable, multi-dimensional metrics grounded in information theory. The use of JSD provides a symmetric, bounded measure of similarity that avoids the limitations of Kullback-Leibler divergence [21, 25], while weighted aggregation enables clinical prioritisation of demographic features. These characteristics make the framework suitable for integration into trial design workflows, where transparency and interpretability are critical.

The weak correlation between cohort size and suitability suggests that representativeness can be achieved even in smaller cohorts. This finding is particularly relevant for early-phase trials, where sample sizes are constrained by feasibility and cost. These results suggest potential utility for trial planning decisions, such as adjusting enrolment strategies to improve demographic alignment or informing documentation for regulatory review, though real-world applicability requires external validation beyond the proof-of-concept scope of this study.

Several limitations warrant consideration. The analysis was based on simulated cohorts derived from a single critical care dataset, which may not capture the full diversity of real-world trial populations. The weighting scheme adopted in this study for the combined metric is illustrative for the purposes of developing the framework, and should be validated further. Robustness checks conducted across alternative feature weight and aggregation weight configurations (Section 3) demonstrated that cohort rankings were stable under aggregation weight perturbation and largely stable under feature weight variation, supporting the reliability of the framework’s comparative assessments. Nevertheless, the absolute suitability scores remain sensitive to the choice of feature weights, and future work should consider empirical or expert-informed approaches to weight derivation, such as the Delphi method [26] or the Best-Worst Method [27], to provide stronger justification for the chosen values. The framework currently addresses demographic representativeness and data completeness, and does not yet incorporate broader clinical dimensions of trial representativeness [28–30]. Extending the approach to include clinical covariates and social determinants of health would provide a more comprehensive assessment. For instance, comorbidity burden (such as diabetes in cardiovascular trials) or frailty indicators (particularly relevant for oncology trials in older adults) can substantially affect treatment response and tolerability, yet are often underrepresented in trial populations. The framework’s treatment of missing demographic data as incomplete could be refined by incorporating fairness-aware methods that account for incompleteness patterns [31]. Practical implementation of this framework requires access to reference population data and modest computational resources. The framework performed reliably across the tested cohort size range (26–84 patients), though validation with smaller (<20 patients) and even larger (>1000 patients), trials warrants future investigation.

While prospective clinical trials typically maintain high demographic data quality, making coverage the dominant driver of suitability, the framework’s explicit completeness assessment becomes particularly valuable when using registry or real-world data as reference populations, where missing data patterns can be substantial. This dual-component approach ensures the framework remains applicable across diverse data quality contexts.

Future work should explore integration with trial design tools, enabling dynamic evaluation of representativeness during protocol development. Applying the framework to multi-centre datasets and diverse therapeutic areas can test its generalisability and inform refinements to weighting strategies. A further direction worth exploring is the use of completeness as a precondition for coverage assessment rather than a parallel metric: features falling below a defined completeness threshold could be flagged or excluded from coverage scoring, ensuring that demographic similarity estimates are not computed from unreliable data. This gatekeeping approach would strengthen the fitness-for-purpose interpretation of the framework and represents a natural extension of the dual completeness construct proposed here.

## 5 Conclusion

This work establishes a quantitative framework for assessing clinical trial cohort representativeness by formalising completeness as a dual construct encompassing both demographic coverage and dataset integrity. By integrating Jensen-Shannon Distance with weighted completeness metrics, the framework transforms representativeness assessment from subjective demographic table comparisons into systematic, reproducible measurements.

Validation using simulated cohorts demonstrates that the framework reliably quantifies trial-population alignment and that coverage and completeness capture independent aspects of data quality. The finding that demographic similarity dominates overall suitability under clinically informed weights reinforces the critical role of representative enrolment in ensuring generalisability. The framework’s explicit treatment of data quality constraints, such as the systematic incompleteness patterns observed in weight and language variables, provides transparency that subjective approaches cannot deliver.

The design of the framework prioritises practical deployment: interpretable metrics support trial design decisions, clinically informed feature weights enable domain-specific customisation, and the modular architecture allows extension to additional demographic dimensions or clinical variables. These characteristics suggest potential for future integration into trial planning workflows, where the approach could inform enrolment strategies, document cohort limitations for regulatory review, and support assessments of completed trials, subject to further validation in real-world settings.

This work demonstrates that representativeness can be measured systematically rather than judged subjectively. As regulatory bodies and journals increasingly mandate demographic reporting, frameworks that provide transparent, quantifiable assessments of trial-population alignment represent a promising direction for supporting evidence-based medicine. This study provides both the methodological foundation and empirical validation required for such systematic assessment, contributing to the broader objective of ensuring clinical research findings translate reliably to real-world treatment populations.

## Data Availability

Publicly available datasets were analyzed in this study. This data can be found here: https://doi.org/10.13026/C2XW26.

## References

[1] Kostis JB Dobrzynski JM. Limitations of randomized clinical trials. Am J Cardiol. (2020) 129:109–15. 10.1016/j.amjcard.2020.05.011 32560898

[2] Mirmiran P Bahadoran Z Gaeini Z. Common limitations and challenges of dietary clinical trials for translation into clinical practices. Int J Endocrinol Metab. (2021) 19:e108170. 10.5812/ijem.108170 34567133 PMC8453651

[3] Rivera SC Liu X Chan A-W Denniston AK Calvert MJ Ashrafian H, et al. Guidelines for clinical trial protocols for interventions involving artificial intelligence: the spirit-AI extension. Lancet Digit Health. (2020) 2:e549–60. 10.1016/S2589-7500(20)30219-3 33328049 PMC8212701

[4] Tetzlaff JM Chan A-W Kitchen J Sampson M Tricco AC Moher D. Guidelines for randomized clinical trial protocol content: a systematic review. Syst Rev. (2012) 1:43. 10.1186/2046-4053-1-43 23006870 PMC3533811

[5] Eshera N Itana H Zhang L Soon G Fadiran EO. Demographics of clinical trials participants in pivotal clinical trials for new molecular entity drugs and biologics approved by FDA from 2010 to 2012. Am J Ther. (2015) 22:435–55. 10.1097/MJT.0000000000000177 25621972

[6] Smith Z Wilkinson M Carney C Grove N Qutab B Getz K. Enhancing the measure of participation burden in protocol design to incorporate logistics, lifestyle, and demographic characteristics. Ther Innov Regul Sci. (2021) 55:1239–49. 10.1007/s43441-021-00336-2 34460095

[7] Staffa SJ Zurakowski D. Five steps to successfully implement and evaluate propensity score matching in clinical research studies. Anesth Analg. (2018) 127:1066–73. 10.1213/ANE.0000000000002787 29324498

[8] Weissler EH Naumann T Andersson T Ranganath R Elemento O Luo Y, et al. The role of machine learning in clinical research: transforming the future of evidence generation. Trials. (2021) 22:537. 10.1186/s13063-021-05489-x 34399832 PMC8365941

[9] Abdullah O Abdel-Qader I Bazuin B. K-means-Jensen–Shannon divergence for a WLAN indoor positioning system. In: *2016 IEEE 7th Annual Ubiquitous Computing, Electronics & Mobile Communication Conference (UEMCON)*. IEEE (2016). p. 1–5. 10.1109/UEMCON.2016.7777924

[10] Li Y-L Wu J-J Ma J Li S-S Xue X Wei D, et al. Alteration of the individual metabolic network of the brain based on Jensen–Shannon divergence similarity estimation in elderly patients with type 2 diabetes mellitus. Diabetes. (2022) 71:894–905. 10.2337/db21-0600 35133397

[11] Menéndez ML Pardo JA Pardo L Pardo MC. The Jensen–Shannon divergence. J Franklin Inst. (1997) 334:307–18. 10.1016/S0016-0032(96)00063-4

[12] Smith J Josef C Xie Y Kamaleswaran R. Online critical-state detection of sepsis among ICU patients using Jensen–Shannon divergence. In: *AMIA Annual Symposium Proceedings*. Vol. 2022. American Medical Informatics Association (2023). p. 982. PMC10148343 37128380

[13] Zhu Z Zhang Z Gao X Feng L Chen D Yang Z, et al. Individual brain metabolic connectome indicator based on Jensen–Shannon divergence similarity estimation predicts seizure outcomes of temporal lobe epilepsy. Front Cell Dev Biol. (2022) 9:803800. 10.3389/fcell.2021.803800 35310541 PMC8926031

[14] Kahn MG Raebel MA Glanz JM Riedlinger K Steiner JF. A harmonized data quality assessment terminology and framework for the secondary use of electronic health record data. eGEMs. (2012) 1:18. 10.13063/2327-9214.1244 PMC5051581 27713905

[15] Miller R Chan SHM Whelan H Gregório J. A comparison of data quality frameworks: a review. Big Data Cogn Comput. (2025) 9:93. 10.3390/bdcc9040093

[16] Miller R Whelan H Chrubasik M Whittaker D Duncan P Gregório J. A framework for current and new data quality dimensions: an overview. Data. (2024) 9:151. 10.3390/data9120151

[17] Wilkinson MD Dumontier M Aalbersberg IJ Appleton G Axton M Baak A, et al. The FAIR guiding principles for scientific data management and stewardship. Sci Data. (2016) 3:160018. 10.1038/sdata.2016.18 26978244 PMC4792175

[18] Johnson A Pollard T Mark R. Data from: MIMIC-III clinical database (version 1.4) (2016). 10.13026/C2XW26

[19] Johnson AEW Pollard TJ Shen L Lehman LWH Feng M Ghassemi M, et al. MIMIC-III, a freely accessible critical care database. Sci Data. (2016) 3:160035. 10.1038/sdata.2016.35 27219127 PMC4878278

[20] Gregório J Lemanska A Cieszynski B Perić N Alsuleman M Chrubasik M, et al. An EHR data standardisation pipeline using the MIMIC-III dataset: foundation for a clinical trial data quality assessment. In: *Proceedings of the 19th International Joint Conference on Biomedical Engineering Systems and Technologies (BIOSTEC 2026)*. Vol. 4. SCITEPRESS (2026). p. 88–96.

[21] Virtanen P Gommers R Oliphant TE Haberland M Reddy T Cournapeau D, et al. Data from: SciPy 1.0: fundamental algorithms for scientific computing in Python (2020). 10.1038/s41592-019-0686-2 PMC7056644 32015543

[22] Harrison JE Weber S Jakob R Chute CG. ICD-11: an international classification of diseases for the twenty-first century. BMC Med Inform Decis Mak. (2021) 21:206. 10.1186/s12911-021-01534-6 34753471 PMC8577172

[23] World Health Organization. Data from: ICD-11: International classification of diseases 11th revision (2018)

[24] Zhou H Liu F Gu B Zou X Huang J Wu J, et al. A survey of large language models in medicine: progress, application, and challenge. *arXiv* [Preprint]. *arXiv:2311.05112* (2024). 10.48550/arXiv.2311.05112

[25] Boyd S Vandenberghe L, Convex Optimization. Cambridge: Cambridge University Press (2004). 10.1017/CBO9780511804441

[26] Dalkey N Helmer O. An experimental application of the DELPHI method to the use of experts. Manage Sci. (1963) 9:458–67. 10.1287/mnsc.9.3.458

[27] Rezaei J. Best-worst multi-criteria decision-making method. Omega. (2015) 53:49–57. 10.1016/j.omega.2014.11.009

[28] Cwalina TB Jella TK Manyak GA Kuo A Kamath AF. Is our science representative? a systematic review of racial and ethnic diversity in orthopaedic clinical trials from 2000 to 2020. Clin Orthop Relat Res. (2022) 480:848–58. 10.1097/CORR.0000000000002050 34855650 PMC9007212

[29] Lancker KV Bretz F Dukes O. Covariate adjustment in randomized controlled trials: general concepts and practical considerations. Clin Trials. (2024) 21:399–411. 10.1177/17407745241251568 38825841

[30] Ye T Shao J Yi Y Zhao Q. Toward better practice of covariate adjustment in analyzing randomized clinical trials. J Am Stat Assoc. (2023) 118:2370–82. 10.1080/01621459.2022.2049278

[31] Feng R Calmon F Wang H. Adapting fairness interventions to missing values. Adv Neural Inf Process Syst. (2023) 36:59388–409.

